# Quantitative analysis of the human ovarian carcinoma mitochondrial phosphoproteome

**DOI:** 10.18632/aging.102199

**Published:** 2019-08-22

**Authors:** Na Li, Shehua Qian, Biao Li, Xianquan Zhan

**Affiliations:** 1 Key Laboratory of Cancer Proteomics of Chinese Ministry of Health, Xiangya Hospital, Central South University, Changsha 410008, Hunan, P. R. China; 2 Hunan Engineering Laboratory for Structural Biology and Drug Design, Xiangya Hospital, Central South University, Changsha 410008, Hunan, P. R. China; 3 State Local Joint Engineering Laboratory for Anticancer Drugs, Xiangya Hospital, Central South University, Changsha 410008, Hunan, P. R. China; 4 National Clinical Research Center for Geriatric Disorders, Xiangya Hospital, Central South University, Changsha 410008, Hunan, P. R. China

**Keywords:** ovarian cancer, mitochondria, TiO enrichment _2_, iTRAQ quantitative proteomics, mitochondrial phosphoprotein (mtPP)

## Abstract

To investigate the existence and their potential biological roles of mitochondrial phosphoproteins (mtPPs) in human ovarian carcinoma (OC), mitochondria purified from OC and control tissues were analyzed with TiO_2_ enrichment-based iTRAQ quantitative proteomics. Totally 67 mtPPs with 124 phosphorylation sites were identified, which of them included 48 differential mtPPs (mtDPPs). Eighteen mtPPs were reported previously in OCs, and they were consistent in this study compared to previous literature. GO analysis revealed those mtPPs were involved in multiple cellular processes. PPI network indicated that those mtPPs were correlated mutually, and some mtPPs acted as hub molecules, such as EIF2S2, RPLP0, RPLP2, CFL1, MYH10, HSP90, HSPD1, PSMA3, TMX1, VDAC2, VDAC3, TOMM22, and TOMM20. Totally 32 mtPP-pathway systems (p<0.05) were enriched and clustered into 15 groups, including mitophagy, apoptosis, deubiquitination, signaling by VEGF, RHO-GTPase effectors, mitochondrial protein import, translation initiation, RNA transport, cellular responses to stress, and c-MYC transcriptional activation. Totally 29 mtPPs contained a certain protein domains. Upstream regulation analysis showed that TP53, TGFB1, dexamethasone, and thapsigargin might act as inhibitors, and L-dopa and forskolin might act as activators. This study provided novel insights into mitochondrial protein phosphorylations and their potential roles in OC pathogenesis and offered new biomarker resource for OCs.

## INTRODUCTION

The completion of human genome sequencing has identified about 20,300 human genes [1]. Researchers are always expecting to clarify molecular mechanisms of a human disease at the level of genome [2]. However, protein is the final performer of life activity, and proteome is much more complex than genome. Variations at the levels of DNAs, RNAs, and proteins lead to proteome diversity; and those variations are commonly derived from mutations, splicing, and post-translational modifications (PTMs) [3]. There are about 400-600 PTMs in human body, such as phosphorylation, acetylation, nitration, ubiquitylation, and glycosylation, which is one of main reasons to cause the diversity of proteins, and influences protein structures and functions [4]. High mortality rate is still an important clinical challenge in ovarian carcinoma (OC) patients. It is necessary to develop new reliable biomarkers and effective therapies for OC patients, which has been driven by proteomics [5].

Mitochondria are crucial and multifunctional subcellular organelle, and are associated with multiple cellular activities and diseases, such as metabolic reprogramming of OC cells [6]. Mitochondria are traditionally viewed just as energy metabolism-related organelles and independent regulation system, which currently is going through a revolutionary change [7, 8]. Literature data showed that mitochondria were interacted with other organelles, and associated with many biological processes through different signal transduction pathways, including cell proliferation, energy metabolism, oxidative stress, ROS production, cell apoptosis, cell cycle, immunity process, and autophagy [8, 9]. The discovery of novel biomarkers and mitochondria-targeting treatment has being attracted many researchers in recent years. Some anti-tumor drugs have been reported to target mitochondria, including melatonin inhibitors, 18 beta-glycyrrhetinic acid, temozolomide, pyrimethamine, T-2 toxin, gossypol acetate, paeoniflorin, Yougui pill, and grifolic acid [10].

Phosphorylation is a common PTM that phosphate (PO_4_) group is added to amino acid residues serine (Ser, S), threonine (Thr, T), and tyrosine (Tyr, Y), which promotes a conformational alteration via interacting with other hydrophilic and hydrophobic residues [11]. Dynamically reversible reaction between phosphorylation and dephosphorylation regulates the basic biological functions [12]. The ratio of phosphorylation occurring at, residues S, T, and Y, is about 90%, 10%, and 0.05%, respectively [13]. Even though the abundance of phosphorylation at residue Y is much lower than that at residues S and T, specific tyrosine kinase inhibitors have demonstrated some amazing effects in various cancers [14]. Many phosphoproteomics studies have been performed at the levels of the whole cell or tissue lysates. However, it might be better to understand the essential regulation mechanisms and biological functions of mitochondria with a spatio-temporal resolution through subcellular fractionation [15]. This study chose human OC mitochondrial phosphoproteome to reveal tumorigenesis, which provided ones a novel promising for cancer therapy and prevention. It is important and necessary to annotate mitochondrial phosphoproteome because (i) it helps enrich mitochondrial functions except energy metabolism; (ii) mitochondrial phosphoproteomic data-based interaction networks benefit for in-depth insights into potential mechanisms of a disease; (iii) it helps understand complex phosphorylation pathways at subcellular level or even signal communication between different organelles; and (iv) it is extremely useful to identify anti-tumor drugs targeting mitochondria [16].

**Figure 1:**
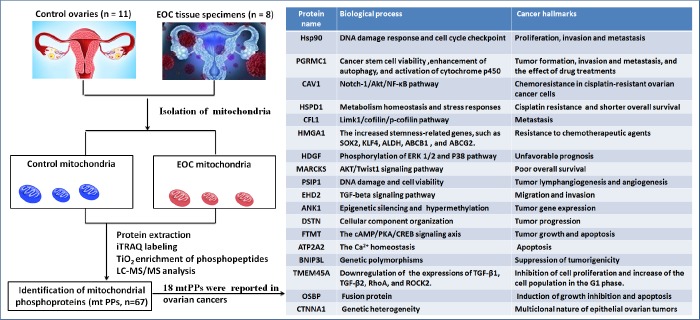
**Experimental flow-chart to identify mtPPs, including 18 previously identified mtPPs.**

This study was the first time to provide further insights into human OC mitochondrial phosphoproteome profiling with iTRAQ quantitative proteomics. A comprehensive analysis was carried out with multiple data, including 18 reported biomarkers, gene ontology, protein-protein interaction networks (PPI), signaling pathways, protein domains, and upstream regulation, which was used to more accurately determine the roles of phosphorylation in OC mitochondria. The current study deepened understanding of the reported biomarkers in OCs, and indicated the relationship of phosphorylation with those molecules. Simultaneously, those non-reported mitochondrial phosphoproteins (mtPPs) might add novel scientific merits for OCs. One should pay more attention to the hub molecules within PPI network, key molecules in enriched pathways, and important phosphorylations within or adjacent to protein domains, which had important scientific value in cell functions, or drug targets in OCs. The experimental flow-chart was used to identify mtPPs and reported biomarkers (Figure 1).

## RESULTS

### Mitochondrial phosphorylation profiling in OCs

**Figure 2:**
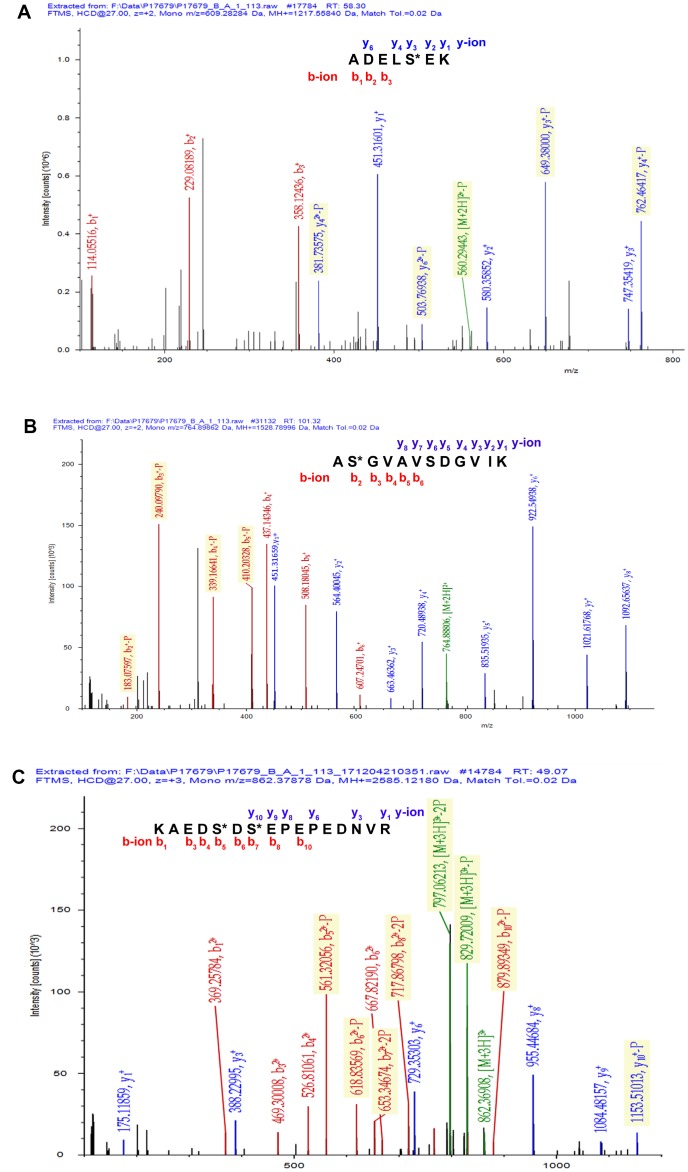
**Representative MS/MS spectra of phosphopeptides.** (**A**) ^2^ADELS*EK^8^ ([M + 2H]^2+^, *m/z* = 609.28284, S* = phosphorylated serine residue) derived from CAV1 (Swiss-Prot No.: C9JKI3). (**B**) ^2^AS*GVAVSDGVIK^13^ ([M + 2H]^2+^, *m/z* = 764.89862, S* = phosphorylated serine residue) derived from CFL1 (Swiss-Prot No.: E9PS23). (**C**) ^419^KAEDS*DS*EPEPEDNVR^434^ ([M + 3H]^3+^, *m/z* = 862.37878, S* = phosphorylated serine residue) derived from XRN2 (Swiss-Prot No.: B4E0B9).

A total of 67 mtPPs based on 85 phosphopeptides with 124 phosphorylation sites were identified in the control and OC samples with iTRAQ-TiO_2_ enrichment-LC-MS/MS between OC mitochondria relative to controls (Supplementary Table 3); and among them, 48 mitiochondrial differentially phosphorylated proteins (mtDPPs) were identified between OC and control samples. A representative MS/MS spectrum was from phosphorylated peptide ^2^ADELS*EK^8^ ([M + 2H]^2+^, *m/z* = 609.28284, S* = phosphorylated serine residue) derived from CAV1 (Swiss-Prot No.: C9JKI3) (Figure 2A), with a high-quality MS/MS spectrum, excellent signal-to-noise (S/N) ratio, and extensive product-ion b-ion and y-ion series (b_1_, b_2_, b_3_, y_1_, y_2_, y_3_, y_4_, and y_6_). The phosphorylation site was localized to amino acid residue S*_6_, and the phosphorylation level was significantly decreased in OCs (T) compared to controls (N) (ratio of T/N = 0.03; p = 8.07E-07) (Supplementary Table 3). Another representative MS/MS spectrum was from phosphorylated peptide ^2^AS*GVAVSDGVIK^13^ ([M + 2H]^2+^, *m/z* = 764.89862, S* = phosphorylated serine residue) derived from CFL1 (Swiss-Prot No.: E9PS23) (Figure 2B), with a high-quality MS/MS spectrum, excellent S/N ratio, and extensive product-ion b-ion and y-ion series (b_2_, b_3_, b_4_, b_5_, b_6_, y_1_, y_2_, y_3_, y_4_, y_5_, y_6_, y_7_, and y_8_). The phosphorylation site was localized to amino acid residue S*_3_, and the phosphorylation level was significantly decreased in OCs compared to controls (ratio of T/N = 0.29; p = 8.25E-05) (Supplementary Table 3). The third representative MS/MS spectrum was from phosphorylated peptide ^419^KAEDS*DS*EPEPEDNVR^434^ ([M + 3H]^3+^, *m/z* = 862.37878, S* = phosphorylated serine residue) derived from XRN2 (Swiss-Prot No.: B4E0B9) (Figure 2C), with a high-quality MS/MS spectrum, excellent S/N ratio, and extensive product-ion b-ion and y-ion series (b_1_, b_3_, b_4_, b_5_, b_6_, b_7_, b_8_, b_10_, y_1_, y_3_, y_6_, y_8_, y_9_, and y_10_). The phosphorylation site was localized to amino acid residues S*_423_ and S*_425_, and the phosphorylation level was not significantly changed in OCs compared to controls (ratio of T/N = 0.86, p = 9.16E-02) (Supplementary Table 3). With the same method, each phosphorylated peptide and phosphorylation site was identified with MS/MS data, and quantified (Supplementary Table 3). Among 67 mtPPs, 18 identified proteins were reported by previous studies in OCs [17], including ANK1, ATP2A2, BNIP3L, CAV1, CFL1, CTNNA1, DSTN, EHD2, FTMT, HDGF, HMGA1, HSP90AA1, HSPD1, MARCKS, OSBP, PGRMC1, PSIP1, and TMEM45A (Figure 1). In terms of mechanism, only CFL1 showed that phosphorylated CFL1 was associated with taxol chemotherapy resistance in human OC cells [18]. The other mtPPs were never reported before. Most of identified phosphorylated sites were occurred at residue Ser, and a few phosphorylated sites were at residues Thr and Tyr. Some mtPPs were phosphorylated at more than one Ser-residues, including BNIP3L, BZW1, DKFZp686M0430, EIF2B5, HMGA1, MYH10, aging-associated protein 14a, OSBP, PDAP1, PKP2, PRKAR2A, PTDSS1, RAB28, RPLP0, STT3B, RPLP2, TOMM20, and XRN2. Some mtPPs were phosphorylated at both residues Ser and Thr, such as PSIP1 (pS_271_, pT_272_, pS_273_, and pS_275_), and PUS1 (pS_127_, and pT_133_). Some mtPPs were phosphorylated at residue Thr, such as KIN27 (pT_179_, and pT_181_). Some mtPPs were phosphorylated at both residues Ser and Tyr, such as NBAS (pS_473_, pS_475_, and pY_477_).

### GO enrichment analysis of mtPPs

**Figure 3:**
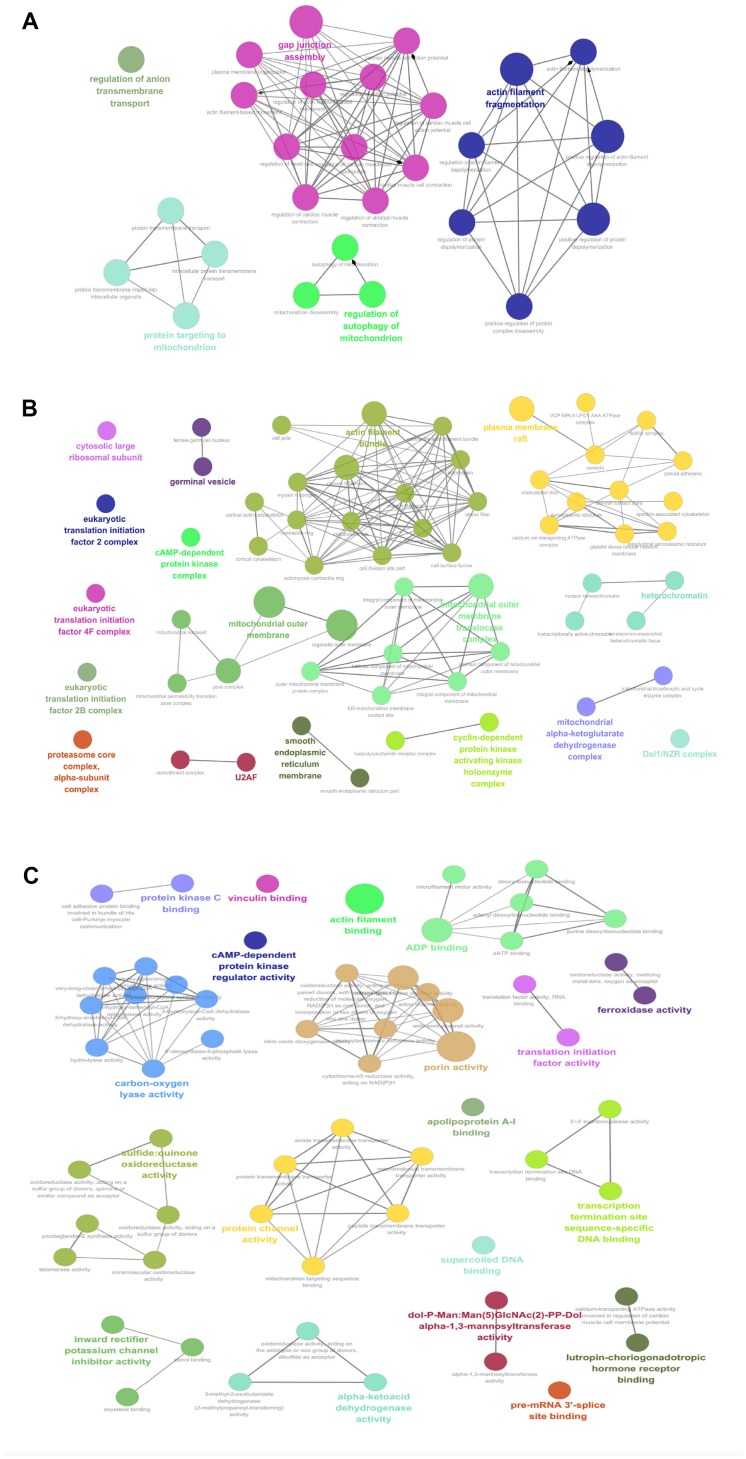
GO analysis revealed biological processes (BPs; **A**), cellular components (CCs; **B**), and molecular functions (MFs; **C**) that involveds in mtPPs.

Those 67 mtPPs between OCs and controls were analyzed with GO enrichment methods, in biological process (BP) (Figure 3A, and Supplementary Table 1), cellular component (CC) (Figure 3B, and Supplementary Table 1), and molecular function (MF) (Figure 3C, and Supplementary Table 1). BP analysis revealed five important biological processes to involve in mtPPs, including anion transmembrane transport, protein targeting to mitochondrion, gap junction assembly, regulation of autophagy of mitochondrion, and actin filament fragmentation. The proteins enriched in the same cluster had biological similarity. CC analysis revealed that mtPPs were mainly involved in cytosolic large ribosomal subunit, eukaryotic translation initiation factor 2B complex, eukaryotic translation initiation factor 4F complex, eukaryotic translation initiation factor 2B complex, proteasome core complex, alpha-subunit complex, germinal vesicle, cAMP-dependent protein kinase regulator activity, U2AF, mitochondrial outer membrane, actin filament bundle, outer mitochondrial membrane protein complex, smooth endoplasmic reticulum part, cyclin-dependent protein kinase activating kinase holoenzyme complex, plasma membrane raft, heterochromatin, mitochondrial alpha-ketoglutarate dehydrogenase complex, and Dsl1/NZR complex. Moreover, some phosphoproteins that were annotated within other cellular compartments but not within mitochondria were also detected in this study, which might be due to interaction with the proteins that are situated at the outer mitochondrial membrane or mitochondria-related proteins. MF analysis revealed that mtPPs were mainly distributed in protein kinase C binding, vinculin binding, actin filament binding, ADP binding, cAMP-dependent protein kinase regulator activity, carbon-oxygen lyase activity, porin activity, translation initiation factor activity, ferroxidase activity, sulfide:quinone oxidoreductase activity, protein channel activity, apolipoprotein A-I binding, transcription termination site sequence-specific DNA binding, supercoiled DNA binding, inward rectifier potassium channel inhibitor activity, alpha-ketoacid dehydrogenase activity, dol-P-Man: Man(5)GlcNAc(2)-PP-Dol alpha-1,3-mannosyltransferase activity, pre-mRNA 3'-splice site binding, and lutropin-choriogonadotropic hormone receptor binding. Some mtPPs also played roles in multiple biological functions.

### PPI network of mtPPs

**Figure 4:**
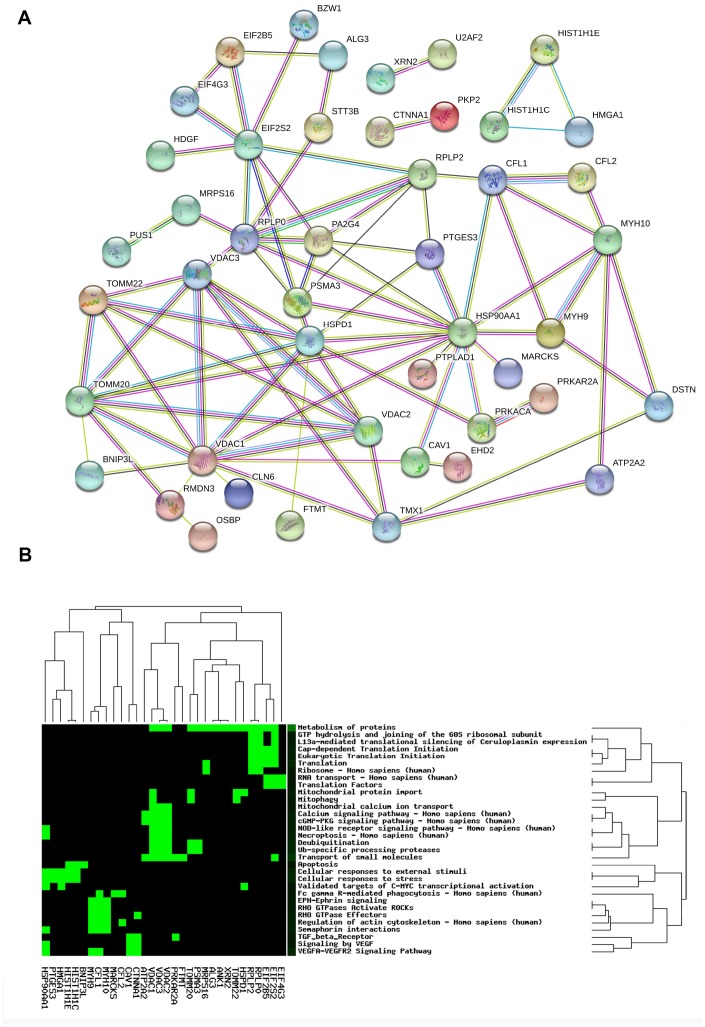
Protein-protein interaction network (**A**) and statistically significant signaling pathways (**B**) involved in mtPPs.

The PPI network of 67 mtPPs was constructed with String analysis database (Figure 4A, and Supplementary Table 2). First, the PPI results revealed 7 mtPP-mtPP pairs had high combined score (value > 0.9) and good correlations (value > 0.4), including RPLP0 and EIF2S2, RPLP0 and MRPS16, DAC2 and VDAC1, RPLP0 and PA2G4, PTGES3 and HSP90AA1, HSPD1 and HSP90AA1, and RPLP0 and RPLP2. Those matched mtPP-mtPP pairs might exist direct or indirect binding sites and co-expression relationship. Second, the PPI results also revealed 20 mtPP-mtPP pairs had high combined score (value>0.9) but low correlations (value>0.4), including CAV1 and HSP90AA1, HSP90AA1 and PRKACA, HMGA1 and HIST1H1E, HMGA1 and HIST1H1C, EIF4G3 and EIF2S2, CFL1 and HSP90AA1, VDAC3 and VDAC2, HSPD1 and TOMM22, MYH10 and MYH9, EIF2S2 and EIF2B5, VDAC3 and VDAC1, VDAC2 and TOMM20, TOMM20 and VDAC1, VDAC3 and TOMM20, HSPD1 and TOMM20, HSPD1 and VDAC1, TOMM20 and TOMM22, EIF2S2 and RPLP2, HIST1H1C and HIST1H1E, and PRKACA and PRKAR2A. The mutual influence between two mtPPs might be though protein-protein binding but not regulating expressions.

### Significant signaling pathways involved in mtPPs

CooLGeN (Version GenCLiP 3) was used to map 67 mtPPs to the corresponding molecules (genes; proteins) for pathway network analysis. A total of 32 statistically significantly signaling pathway systems (p < 0.05) were identified with 67 mtPPs, and were grouped into 15 clusters (Figure 4B, and Table 1). Cluster 1 included mitophagy, and mitochondrial protein import pathways. Cluster 2 included apoptosis pathway. Cluster 3 included Ub-specific processing proteases, and deubiquitination pathways. Cluster 4 included vascular endothelial growth factor (VEGF), and TGF beta receptor signaling. Cluster 5 included RHO GTPases-activated ROCKs, EPH-Ephrin signaling, semaphoring interactions, regulation of actin cytoskeleton, and RHO GTPase effectors. Cluster 6 included VEGFA VEGFR2 signaling pathway. Cluster 7 included mitochondrial calcium ion transport. Cluster 8 included eukaryotic translation initiation, cap dependent translation initiation, translation, and ribosome. Cluster 9 included necroptosis, and NOD-like receptor signaling pathway. Cluster 10 included translation factors, and RNA transport. Cluster 11 included cGMP-PKG signaling pathway, calcium signaling pathway, and transport of small molecules. Cluster 12 included metabolism of proteins. Cluster 13 included the validated targets of C-MYC transcriptional activation, cellular responses to external stimuli, and cellular responses to stress. Cluster 14 included Fc gamma R-mediated phagocytosis. Cluster 15 included L13a-mediated translational silencing of ceruloplasmin expression, and GTP hydrolysis and joining of the 60S ribosomal subunit.

**Table 1:** Classification of the identified phosphoproteins into 15 clusters according to the KEGG pathway analysis.

**Pathway name**	**P-value**	**Q-value**	**Gene list**	**Database**
***Cluster 1***				
Mitophagy	2.51E-04	2.06E-03	TOMM20; TOMM22; VDAC1	Reactome
Mitochondrial_protein import	1.47E-04	1.81E-03	HSPD1; TOMM20; TOMM22; VDAC1	Reactome
***Cluster 2***				
Apoptosis	5.55E-03	1.32E-02	BNIP3L; HIST1H1C; HIST1H1E	Wikipathways
***Cluster 3***				
Ub-specific_processing proteases	2.10E-03	9.13E-03	PSMA3; TOMM20; VDAC1; VDAC2; VDAC3	Reactome
Deubiquitination	2.69E-03	9.94E-03	PSMA3; TOMM20; VDAC1; VDAC2; VDAC3	Reactome
***Cluster 4***				
Signaling_by VEGF	8.15E-03	1.72E-02	CAV1; CTNNA1; HSP90AA1	Reactome
TGF_beta Receptor	3.51E-02	5.31E-02	CAV1; CTNNA1; PRKAR2A	NetPath
***Cluster 5***				
RHO_GTPases Activate ROCKs	6.18E-05	2.29E-03	CFL1; MYH10; MYH9	Reactome
EPH-Ephrin_signaling	3.52E-03	1.09E-02	CFL1; MYH10; MYH9	Reactome
Semaphorin_interactions	1.38E-04	2.04E-03	CFL1; HSP90AA1; MYH10; MYH9	Reactome
Regulation_of Actin Cytoskeleton	2.45E-02	4.11E-02	CFL1; CFL2; MYH10	Wikipathways
RHO_GTPase Effectors	4.04E-02	5.98E-02	CFL1; CTNNA1; MYH10; MYH9	Reactome
***Cluster 6***				
VEGFA-VEGFR2_Signaling Pathway	2.84E-03	9.56E-03	CAV1; CFL1; CTNNA1; HSP90AA1; MYH9	Wikipathways
***Cluster 7***				
Mitochondrial_calcium ion transport	7.25E-05	1.79E-03	VDAC1; VDAC2; VDAC3	Reactome
***Cluster 8***				
Eukaryotic_Translation Initiation	2.19E-03	9.02E-03	EIF2B5; EIF2S2; RPLP0; RPLP2	Reactome
Cap-dependent_Translation Initiation	2.19E-03	8.54E-03	EIF2B5; EIF2S2; RPLP0; RPLP2	Reactome
Translation	3.94E-03	1.12E-02	EIF2B5; EIF2S2; MRPS16; RPLP0; RPLP2	Reactome
Ribosome_- Homo sapiens (human)	2.53E-02	4.16E-02	MRPS16; RPLP0; RPLP2	KEGG
***Cluster 9***				
Necroptosis - Homo sapiens (human)	4.46E-03	1.22E-02	HSP90AA1; VDAC1; VDAC2; VDAC3	KEGG
NOD-like_receptor signaling pathway - Homo sapiens (human)	5.07E-03	1.29E-02	HSP90AA1; VDAC1; VDAC2; VDAC3	KEGG
***Cluster 10***				
Translation_Factors	1.14E-03	5.63E-03	EIF2B5; EIF2S2; EIF4G3	Wikipathways
RNA_transport - Homo sapiens (human)	3.36E-02	5.18E-02	EIF2B5; EIF2S2; EIF4G3	KEGG
***Cluster 11***				
cGMP-PKG_signaling pathway - Homo sapiens (human)	4.56E-03	1.20E-02	ATP2A2; VDAC1; VDAC2; VDAC3	KEGG
Calcium_signaling pathway - Homo sapiens (human)	7.24E-03	1.58E-02	ATP2A2; VDAC1; VDAC2; VDAC3	KEGG
Transport_of small molecules	2.02E-02	3.65E-02	ATP2A2; EIF2S2; FTMT; PRKAR2A; VDAC1; VDAC2; VDAC3	Reactome
***Cluster 12***				
Metabolism_of proteins	1.14E-02	2.27E-02	ALG3; ANK1; EIF2B5; EIF2S2; HSPD1; MRPS16; PSMA3; RPLP0; RPLP2; TOMM20; TOMM22; VDAC1; VDAC2; VDAC3; XRN2	Reactome
***Cluster 13***				
Validated_targets of C-MYC transcriptional activation	5.55E-03	1.37E-02	HMGA1; HSP90AA1; HSPD1	PID
Cellular_responses to external stimuli	3.00E-02	4.72E-02	HIST1H1C; HIST1H1E; HMGA1; HSP90AA1; PTGES3	Reactome
Cellular_responses to stress	8.97E-03	1.84E-02	HIST1H1C; HIST1H1E; HMGA1; HSP90AA1; PTGES3	Reactome
***Cluster 14***				
Fc_gamma R-mediated phagocytosis - Homo sapiens (human)	6.28E-03	1.45E-02	CFL1; CFL2; MARCKS	KEGG
***Cluster 15***				
L13a-mediated_translational silencing of Ceruloplasmin expression	1.49E-02	2.83E-02	EIF2S2; RPLP0; RPLP2	Reactome
GTP_hydrolysis and joining of the 60S ribosomal subunit	1.52E-02	2.81E-02	EIF2S2; RPLP0; RPLP2	Reactome

When mitochondria were in the status of imbalance, such as stress or damage, defective mitochondria might enter selective degradation process by autophagy, named mitophagy. Mitophagy is a crucial mechanism of quality control in mitochondria and can be regarded as a double-edged sword in cancer cells. The defective mitochondria that responded to oncogenic stresses could either stimulate or block tumorigenesis through mitophagy [19]. Here, the new biomarkers that were found in the mitophagy pathway included phosphorylations of TOMM20 at residues S_135_ and S_138_ (fold change = 3.54, p = 1.00E-04), TOMM20 at residue S_135_ (fold change = 2.56, p = 8.00E-05), TOMM22 at residue S_15_ (fold change = 1.16, p = 1.00E-05), and VDAC1 at residue S_104_ (fold change = 2.63, p = 1.00E-04), which might be novel therapeutic targets and fundamental improvement of the damaged mitochondria in OC patients. These data showed that mitophagy might play important role in mediating tumorigenesis and tumor progression.

VEGF signaling is related to many physiological and pathological processes in human. VEGF is a specific heparin-binding growth factor of vascular endothelial cells, which can induce angiogenesis *in vivo*. At the same time, VEGF can increase vascular permeability and mobility of cancer cells, induce tumor angiogenesis, and maintain the growth of tumors [20]. Here, the new biomarkers that were found in the VEGF signaling included phosphorylations of CAV1 at residue S_6_ (fold change = 0.03, p = 8.07E-07), CAV1 at residue S_26_ (fold change = 0.06, p = 9.35E-09), CTNNA1 at residue S_518_ (fold change = 0.78, p = 1.00E-04), HSP90 at residue S_217_ (fold change = 0.80, p =2.83E-02), CFL1 at residue S_3_ (fold change = 0.29, p = 8.00E-05), and MYH9 at residue S_1943_ (fold change = 1.40, p = 7.70E-03), which might be novel molecules and regulation mechanism for tumor angiogenesis.

Rho GTPases are the members of small GTPases, and Rho GTPase pathway is closely related to cytoskeleton reorganization, cell morphology, apoptosis, cell migration, and cell cycle. Recent studies showed that Rho GTPases mediated tumor invasion and metastasis by regulating extracellular matrix remodeling, loss of epithelial polarity, lymphatic vasculature, cytoskeleton reorganization, and between-cell junction and adhesion [21]. Here, the new biomarkers that were found in Rho GTPase signaling included phosphorylations of CFL1 at residue S_3_ (fold change = 0.29, p = 8.00E-05), MYH10 at residue S_1975_ (fold change = 0.82, p = 3.80E-01), MYH10 at residue S_1956_ (fold change = 0.80, p = 3.68E-01), MYH10 at residues S_1975_ and S_1956_ (fold change = 1.77, p = 3.99E-02), MYH9 at residue S_1943_ (fold change = 1.40, p = 7.70E-03), and CTNNA1 at residue S_518_ (fold change = 0.78, p = 1.00E-04), which might be novel molecules and regulation mechanism for tumor invasion and metastasis.

Multiple signal transduction pathways are initiated by calcium ions (Ca^2+^), so intracellular calcium ions are the necessary condition for occurrence, development, and proliferation of tumors through various pathways [22]. Mitochondrial calcium ion transport pathway is important in regulating Ca^2+^ concentrations in cells. This study found that mitochondrial calcium ion transport pathway and calcium signaling pathway were enriched to involve mtPPs, including phosphorylations of VDAC1 at residue S_104_ (fold change = 2.63, p = 1.00E-04), VDAC2 at residue S_140_ (fold change = 1.99, p = 7.90E-03), VDAC3 at residue T_6_, and ATP2A2 at residue S_554_ (fold change = 0.69, p = 2.39E-02). Imbalance of Ca^2+^ might be the cause of tumorigenesis, so it is definitely worth further exploring.

C-Myc-mediated transcriptional activity promotes tumorigenesis and development. The major oncogenic mechanisms of c-MYC are related to promotion of cell cycle, drug resistance, reprogramming of cell metabolism, and rapid proliferation [23]. Here, the new biomarkers that were found in C-MYC-mediated transcriptional activation pathway included phosphorylations of HMGA1 at residues S_92_ and S_93_ (fold change = 2.02, p = 1.70E-03), and at residues S_88_, S_92_, and S_93_, HSP90 at residue S_217_ (fold change = 0.80, p = 2.83E-02), and HSPD1 at residue S_70_ (fold change = 2.34, p = 3.40E-03). Enhanced c-Myc-mediated transcriptional activity might induce cancer.

### Relationships of phosphorylated sites and functional domains in mtPPs

**Figure 5:**
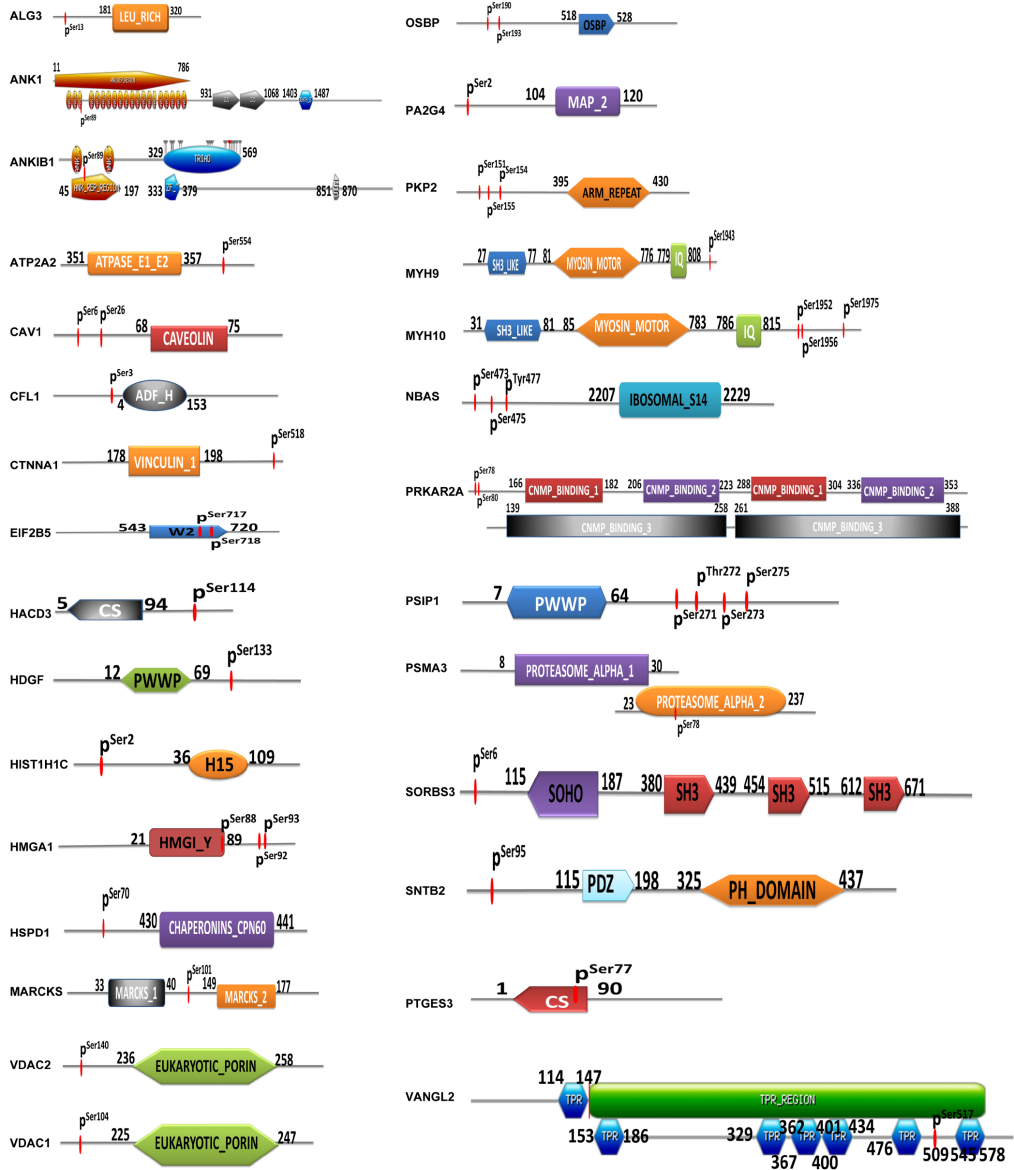
**Identification of phosphorylated sites and protein domains in mtPPs.**

A total of 29 identified mtPPs contained a certain structural and functional domains (Figure 5). Conjoint analysis of protein domains of mtPPs provided novel insights into mitochondrial protein phosphorylation and its potential role in OCs. First, this study found that ANK1, ANKIB1, EIF2B5, HMGA1, CS, PSMA3, and VANGL2 had phosphorylation sites within protein domains. ANK1 contained ankyrin-repeated region circular (sites: 11-786), with phosphorylation at residue S_89_. ANKIB1 also contained ankyrin-repeated region circular (sites: 45-197), with phosphorylation at residue S_89_. EIF2B5 contained W2 domain (sites: 543-720), with phosphorylation at residues S_717_ and S_718_. HMGA1 contained HMGI_Y domain (sites: 21-89), with phosphorylation at residue S_88_. PSMA3 contained proteasome alpha domain (sites: 8-30 and 23-237), with phosphorylation at residue S_78_. VANGL2 contained TPR regions (sites: 114-578), with phosphorylation at S_517_. Second, CFL1 and HMGA1 were phosphorylated near protein domains. CFL1 contained actin-depolymerising factor homology domain (ADF-H domain) (sites: 4-153), with phosphorylation at residue S_3_. HMGA1 contained HMG-Y DNA-binding domain (AT-hooks) (sites: 21-89), with phosphorylation at residues S_92_ and S_93_. Third, the phosphorylation sites of the other 20 mtPPs with structural and functional domains were neither presented within domains nor near domains. However, it doesn't mean that those mtPPs were not important. The between-molecule abscopal effects could not been ignored. Additionally, phosphorylation at residue Y was a low abundance event compared to that at residues S and T. Specific tyrosine kinase inhibitors had been discovered to have some pretty amazing therapeutic effects in different types of cancers. NBAS contained ribosomal protein S14 signature (sites: 2207-2229), with phosphorylations at residues S_473_, S_475_ and Y_477_. This protein was associated with transporting proteins from Golgi complex to endoplasmic reticulum.

### Upstream analysis indicated potential regulatory mechanism

**Figure 6:**
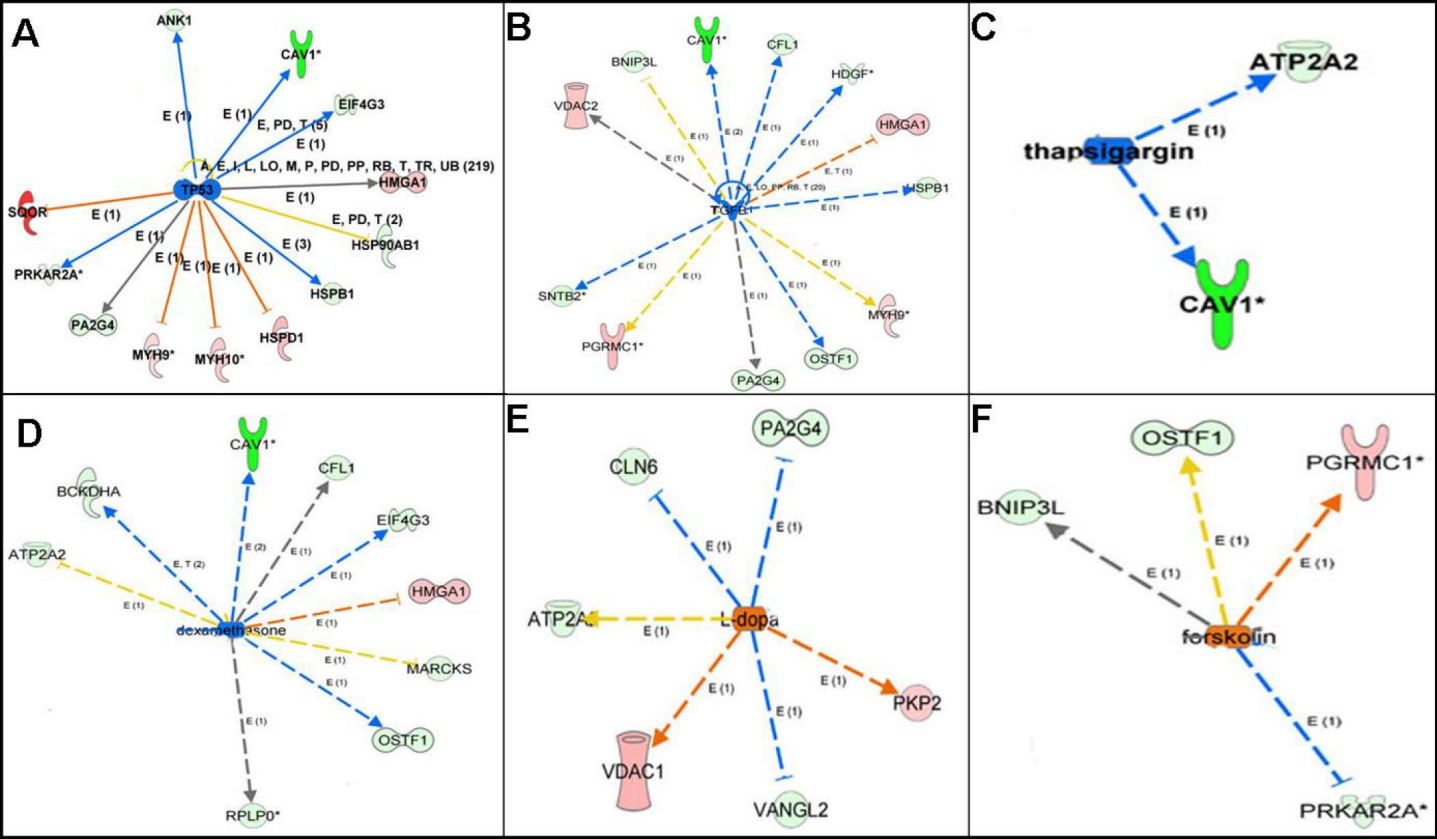
**Upstream regulation analysis revealed the upstream regulators of mtPPs.** Note: The solid line means direct interaction. The dotted line means indirect interaction. The arrow line means activation. The non-arrow line means inactivation. The red molecule means increased measurement. The green molecule means decreased measurement.

Upstream regulation analysis of 67 mtPPs indicated potential regulatory mechanisms and provided potential anti-cancer drugs (Figure 6 and Table 2). Upstream regulation analysis showed that TP53 might be an inhibitor for PRKAR2A, MYH9, MYH10, HSPD1, EIF4G3, ANK1, HSP90AB1, HMGA1, and PA2G4. In human, transforming growth factor beta 1 (TGF-β1) was encoded by TGFB1 gene, which was a secreted protein. Upstream regulation analysis showed that TGFB1 might be an inhibitor for PGRMC1, HMGA1, VDAC2, MYH9, OSTF1, HDGF, BNIP3L, PA2G4, CFL1, and SNTB2. Dexamethasone was a kind of corticosteroid medication. Upstream regulation analysis showed that dexamethasone might be an inhibitor for HMGA1, OSTF1, ATP2A2, BCKDHA, MARCKS, EIF4G3, CFL1, and RPLP0. Thapsigargin, as a tumor promoter in mammalian cells, was non-competitive inhibitor of sarcoplasmic and endoplasmic reticulum Ca^2+^ ATPase. Upstream regulation analysis showed that dexamethasone might be an inhibitor for ATP2A2, and CAV1. L-dopa was also known as L-3,4-dihydroxyphenylalanine. Upstream regulation analysis showed that L-dopa might be an activator for VDAC1, PKP2, CLN6, VANGL2, ATP2A2, and PA2G4. Forskolin was a commonly used chemical toxicant in laboratory to increase cyclic AMP level. Upstream regulation analysis showed that forskolin might be an activator for PGRMC1, OSTF1, BNIP3L, and PRKAR2A.

**Table 2:** Upstream regulation analysis revealed the upsream regulators that were involved in identified phosphoproteins.

**Upstream regulator**	**Molecule type**	**Predicted function**	**Activation score**	**p-value**	**Target molecules**
TP53	transcription regulator	inhibited	−2.076	0.0000094	PRKAR2A; MYH9; MYH10; HSPD1; EIF4G3; ANK1; HSP90AB1; HMGA1; PA2G4
TGFB1	growth factor	inhibited	−1.928	0.0000333	PGRMC1; HMGA1; VDAC2; MYH9; OSTF1; HDGF; BNIP3L; PA2G4; CFL1; SNTB2
Dexamethasone	chemical drug	inhibited	−1.947	0.0000759	HMGA1; OSTF1; ATP2A2; BCKDHA; MARCKS; EIF4G3; CFL1; RPLP0
Thapsigargin	chemical toxicant	inhibited	−1.982	0.000325	ATP2A2; CAV1
L-dopa	chemical-endogenous molecule	activited	1.937	0.000893	VDAC1; PKP2; CLN6; VANGL2; ATP2A2; PA2G4
Forskolin	chemical toxicant	activited	1.664	0.0000594	PGRMC1; OSTF1; BNIP3L; PRKAR2A

## DISCUSSION

Protein phosphorylation regulates multiple cellular processes through complex and highly dynamic signaling pathways. Multiple PTMs are one of the reasons to form proteoforms that are ultimate actors for cellular activity. Distinguishing of different proteoforms benefits for real precision medicine practice [24]. In cancer, the changed phosphorylation in a protein is found to be closely correlated with tumorigenesis and influence malignant progression through multiple biological processes, such as tumor angiogenesis, cell cycle, energy metabolism, apoptosis, tumor immunity, and cell proliferation. The previous study showed that clinical significance and subcellular distribution of protein phosphorylation were associated with poorly differentiated and high tumor node metastasis (TNM) stage tumors [25]. Multivariate survival analysis even suggested that the concentration of phosphorylated protein was independent prognostic factor for carcinoma [26]. Recently, protein-protein interaction and functional enrichment analysis revealed that phosphorylation network pathways and location of phosphoproteins in subcellular compartment had specific and dynamic characteristics [27]. Large-scale comparative phosphoproteomics studies on whole cells or subcellular organelles were frequently done with MS approaches. The previous phosphoproteomics found that phosphorylations of YES at multiple residue sites within its N-terminal unique domain were significantly increased in highly recurrent OC patients, and that YES phosphorylations affected expressions of multiple cell-cycle regulators, which suggested YES as a potential target for the treatment of cancer [28]. Recent comprehensive proteogenomic analysis of OC tissues revealed that autophosphorylation status of PTK2 (pY_397_) and PTK2B (pY_402_) was altered in tumor tissues [29]. Taken together, previous studies demonstrated that phosphorylation profile facilitated to understand the dynamics of cancer-related pathways, and their roles in disease processes.

Every subcellular component is highly dynamic because of their PTMs, abundance, and the protein expressions depending on cell physiological state. Therefore, it is very important to identify subcellular phosphoproteins for understanding biological functions of proteins in specific compartment. Mitochondria are classically viewed as isolated, spherical, and energy metabolism-related organelles, which is going through a revolutionary change. Literature data showed that mitochondria were associated with energy metabolism, oxidative stress, cell apoptosis, cell cycle, autophagy, and immunity process. In fact, mitochondrial pathways were involved in many diseases, including malignant tumors. For example, high levels of AKAP1 were found in variety of high-grade cancer tissues. AKAP1 scaffolding protein integrated Src and cAMP signaling on mitochondria to regulated oxidative metabolism, organelle biogenesis, and cell survival [30]. These findings promoted mitochondrial proteome to become a research hotspot. To investigate the proteomic profile of OC mitochondrial proteins, iTRAQ quantitative proteomics was used to identify mitochondrial proteins expressed in OC tissues relative to controls in our recent study [5]. The increasing number of mitochondrial phosphoproteins and phosphorylation sites identified was mainly ascribed to recent advances in phosphoproteomic technologies such as fractionation, phosphopeptide enrichment, and high-sensitivity MS. However, one must determine the functional importance of specific kinases and phosphatases in regulation of phosphorylation-dephosphorylation process for these identified mitochondrial phosphorylation sites [16]. This study identified 67 mtPPs in OCs relative to controls, which of them, 18 mtPPs were already reported in other literature, including HSP90, PGRMC1, CAV1, HSPD1, CFL1, HMGA1, HDGF, MARCKS, PSIP1, EHD2, ANK1, DSTN, FTMT, ATP2A2, BNIP3L, TMEM45A, OSBP, and CTNNA1. It clearly demonstrated that iTRAQ quantitative proteomics was a reliable method to identify mtPPs and their phosphorylation sites. Studies showed the influence of altered phosphorylation on the malignant phenotype of tumor cells. For example, the prominently increased phosphorylation of cofilin-1 at residue S_3_ had a significant effect on glutamine uptake by cells, which facilitated nuclear translocation, and changed actin organization [31]. Blockage of phosphorylation of Cav-1 eliminated translocation of β-catenin from cytomembrane to cytoplasm [32]. Compared to previous literature, just phosphorylations of MARCKS and CAV1 identified in this study were already reported in ovary diseases. Decreased phosphorylation of CAV1 at residue Y_14_ was related to the insulin-resistant state in endometrial tissue of polycystic ovary syndrome patients. Plasma membrane was required for phosphorylation of membrane-associated MARCKS to reduce the amount of F-actin [33]. A total of 49 mtPPs identified in this study were not reported in previous literature based on CooLGeN database (http://ci.smu.edu.cn/CooLGeN/Home.php). These new identified mtPPs and their phosphorylation sites might be malignancy-associated intracellular events in OCs.

A total of 32 statistically significant pathways (p<0.05) was identified to involve mtPPs, which associated closely with the occurrence of cancer to indicate potential molecular mechanisms that mtPPs played in OCs. Additionally, a total of 29 identified mtPPs contained a certain structural and functional domains. This study provided novel insights into phosphorylations of mitochondrial proteins and their potential roles in contribution to molecular mechanisms of an OC. ANK1, ANKIB1, EIF2B5, HMGA1, CS, PSMA3, and VANGL2 were phosphorylated within protein domains. A certain structural and functional domains played important roles in tumor biological behaviors. For example, ANK1 contained ankyrin-repeated region cicular. Ankyrins, which link the membrane proteins to the spectrin-actin cytoskeleton, were encoded by ANK1 gene, and played crucial roles in multiple cellular activities such as proliferation, motility, and membrane domain activation. The 24 tandem ankyrin repeats could recognize a wide range of membrane proteins through non-specific interactions, such as hydrogen bonding, electrostatic interactions, and hydrophobic interactions [34]. EIF2B5 contained W2 domain. EIF2B5 gene encoded subunits of eukaryotic translation initiation factor 2B as an essential regulator for protein synthesis as a GTP exchange factor. The W2 domain contained aromatic/acidic residue-rich regions and was important for protein-protein interactions [35]. VANGL2 contained TPR-repeat region circular. The protein encoded by VANGL2 gene was related to the regulation of planar cell polarity and transmitted directional signals to groups of cells or individual cells in epithelial sheets. The TPR domains facilitated specific interactions with certain partner proteins. Most TPR-containing proteins involved in cell-cycle, formation of multiprotein complexes, and transcription [36]. CFL1 and HMGA1 were phosphorylated near protein domains. CFL1 contained actin-depolymerising factor homology domain (ADF-H domain). CFL1 protein polymerized and depolymerized G-actin and F-actin in a pH-dependent manner, which was involved in the transfer of actin-cofilin complex from cytoplasm to nucleus. ADF-H domain was mainly responsible for interactions with actin with high affinity [37]. HMGA1 contained HMG-I and HMG-Y DNA-binding domain (AT-hooks). HMGA1 protein was involved in regulation of DNA replication, integration of retroviruses into chromosomes, gene transcription, and metastatic progression of cancer cells. AT-hooks, included the RGRP (Arg-Gly-Arg-Pro) core motif, showed high affinity to AT-rich DNA *in vitro*, which provided a clue to high amounts of diverse PTMs of HMGA1 protein [38].

Furthermore, upstream regulation analysis showed that TP53, TGFB1, dexamethasone, and thapsigargin might act as inhibitors, and that L-dopa, and forskolin might act as activators. TP53 gene was one of the most frequently mutated genes (>50%) in human cancers, it might play a crucial role in cancer formation. TP53 gene also encoded a certain proteins that bond to DNA to prevent mutations of genome. In human, the secreted protein TGF-β1 was encoded by TGFB1 gene. TGF-β1 performed various cellular functions, including cell proliferation, cell growth, apoptosis, and cell differentiation. Upstream regulation analysis showed that dexamethasone might be an inhibitor for ATP2A2 and VCL. Dexamethasone, a kind of corticosteroid medication, was one of essential medicines in the World Health Organization's List, which was usually given to counteract a certain side-effects for cancer patients. Actually, dexamethasone was also used as a chemotherapeutic agent in some malignancies, in which it was given alone or combined with other chemotherapeutic drugs such as lenalidomide, thalidomide, vincristine, bortezomib or doxorubicin [39]. Thapsigargin, as a tumor promoter in mammalian cells, was a non-competitive inhibitor of sarcoplasmic and endoplasmic reticulum Ca^2+^-ATPases, which influenced intracellular calcium concentration by blocking cell capacity to pump calcium into the sarco/endoplasmic reticular [40]. L-dopa, namely L-3,4-dihydroxyphenylalanine, was an effective drug for dopamine-responsive dystonia or Parkinson's disease, which was tested as an effective anti-cancer agent in mice and selectively inhibited the growth of human melanoma cells, although no evidence was found for L-dopa as antitumor activity *in vivo* [41]. Forskolin was a commonly used chemical toxicant in laboratory to increase cyclic AMP level by stimulating adenylate cyclase; however, studies found that cAMP pathway and an abnormal activation of cAMP-regulated genes were related to cancer growth [42]. Therefore, upstream regulation analysis clearly revealed potential regulatory mechanism and provided potential anti-cancer drugs for OCs.

This study is the first report to investigate globally mitochondrial protein phosphorylations and their potential biological roles in the pathological processes of human OCs with iTRAQ-labeled TiO_2_ enrichment-LC-MS/MS method. Many identified mtPPs and their phosphorylation sites have not been reported previously, which benefit for the discovery of novel biomarkers to clarify basic molecular mechanisms of human OC formation, and truly predict OC progression.

## MATERIALS AND METHODS

### Cancer and control tissues

Frozen OC tissues (n = 8) and control ovaries with benign gynecologic disease (n = 11) were obtained from Department of Gynecology, Xiangya Hospital, Central South University, China. This study was approved by the Medical Ethics Committee of Xiangya Hospital, and the written informed consent was obtained from each patient. Both OC and control tissues were verified with histological analysis. Each tissue sample was immediately placed in liquid nitrogen and then stored at −80°C.

### Preparation of mitochondrial proteins

Ovarian tissue samples were fully minced in pieces and homogenized in mitochondrial isolation buffer that contained Nagarse, followed by differential-speed centrifugation to obtain crude mitochondria. The crude mitochondria were further purified with Nycodenz-gradient centrifugation. The purified mitochondria samples were verified by western blot and electron microscopy. The detailed experimental procedure was described previously [4, 5]. The prepared OC and control mitochondria samples were used to extracted proteins with SDT lysis buffer that contained 4% SDS, 1 mM DTT, and 100 mM Tris-HCl pH 7.6, followed by centrifugation (14,000 g, 40 min) to collect the supernatant as extracted mitochondrial proteins. The protein content was quantified with the BCA Protein Assay Kit (Bio-Rad, USA).

### iTRAQ labeling

An amount (200 μg) of extracted mitochondrial proteins were mixed with 30 μl solution that contained 4% SDS, 100 mM DTT, and 150 mM Tris-HCl pH 8.0. The detergent, DTT, and other low-molecular-weight components were removed by repeated ultrafiltration with the solution of 8 M urea and 150 mM Tris-HCl pH 8.0. The reduced cysteine residues were blocked with adding 100 μl solution that contained 100 mM iodoacetamide, 8 M urea, and 150 mM Tris-HCl pH 8.0, for incubation in darkness for 30 min, followed by washing with 100 μl solution (8 M urea and 150 mM Tris-HCl pH 8.0) for 3 times and then 100 μl dissolution buffer for 2 times. The protein suspensions were digested with 4 μg trypsin (Promega) (37 °C, overnight). The tryptic peptides were desalted on C18 Cartridges, concentrated by vacuum centrifugation. The peptide content was estimated by UV light spectral density at 280 nm. A amount (100 μg) of tryptic peptide mixture of each sample was labeled using iTRAQ reagent. Each sample was labeled by three different iTRAQ reagents. The detailed experimental procedure was described previously [4, 5].

### Enrichment of phosphopeptides

Six iTRAQ-labeled peptides were equally mixed, concentrated by a vacuum concentrator, and resuspended in 500 μl DHB buffer. TiO_2_ beads were added and agitated for 2 h, followed by centrifugation (1 min, 5000 g) to keep the beads, washing with 50 μL of washing buffer I (30% acetonitrile and 3% trifluoroacetic acid) (3 x) and then 50 μL of washing buffer II (80% acetonitrile and 0.3% trifuoroacetic acid) (3 x) to remove the remaining non-adsorbed material. Finally, phosphopeptides were eluted with 50 μL of elution buffer (40% acetonitrile and 15% NH_3_·H_2_O) (3 x), and then lyophilized.

### LC-MS/MS of enriched phosphopeptides

The TiO_2_-enriched phosphopeptides were analyzed with LC-MS/MS. Briefly, the enriched phosphopeptides were loaded onto a reverse phase trap column (Thermo Scientific Acclaim PepMap100, 100 μm x 2 cm, nanoViper C18) connected to the C18-reversed phase analytical column (Thermo Scientific Easy Column, 10 cm long, 75 μm inner diameter, 3 μm resin) in buffer A (0.1% formic acid) and separated with a linear gradient of buffer B (84% acetonitrile and 0.1% formic acid) at a flow rate of 300 nl/min controlled by IntelliFlow technology. The linear gradient was 0–55% buffer B for 220 min, 55-100% buffer B for 8 min, and then 100% buffer B for 12 min. MS/MS analysis was performed on a Q Exactive mass spectrometer (Thermo Scientific), with the following parameters, including positive ion mode, data-dependent top10 method to choose the most abundant precursor ions from the survey scan (300–1800 m/z) for HCD fragmentation, automatic gain control (AGC) target 3e6, and maximum inject time to 10 ms, dynamic exclusion duration 40.0 s, resolution for MS set to 70,000 at *m/z* 200, resolution for MS/MS set to 17,500 at *m/z* 200, isolation width set to 2 *m/z*, normalized collision energy set to 30 eV, the underfill ratio set to 0.1%. MS/MS spectra were used to search protein database with MASCOT engine (Matrix Science, London, UK; version 2.2) embedded into Proteome Discoverer 1.4. The intensities of iTRAQ reporter ions were used to determine differentially phosphorylated proteins between OC and control mitochondrial samples.

### Bioinformatics analysis

The reported biomarkers for OC based on the identified mtPPs were obtained from NCBI (https://www.ncbi.nlm.nih.gov/pubmed/). Gene ontology, including BP and CC, was analyzed with Cytoscape ClueGO to obtain more insights into the biological information of mtPPs. PPI network was analyzed with String database (http://string-db.org/cgi/input.pl). Pathway enrichment was analyzed by CooLGeN (http://ci.smu.edu.cn/CooLGeN/Home.php) based on Reactome, Wikipathways, NetPath, and KEGG database. Each MS/MS-derived phosphoprotein amino acid sequence was input into the ScanProsite program (http://prosite.expasy.org/scanprosite) to determine its protein domains and relationship of phosphorylation sites and protein domains. The Swiss-Prot accession numbers and corresponding fold-changes between OCs and controls were input to the Ingenuity Pathway Analysis (IPA) data-upload workflow. The upstream regulation analysis was generated to involve those phosphoproteins.

### Statistical analysis

The Student’s *t-*test was used to assess mtDPPs between OC and control groups. P-value for GO enrichment analysis was obtained by two-sided hypergeometric test and corrected by Benjamini-Hochberg. P-value for pathway enrichment analysis was obtained by two-sided hypergeometric test and corrected by Q-value. The level of statistical significance was set as p < 0.05.

### Ethical approval

All the patients were informed about the purposes of the study and consequently have signed their “consent of the patient”. All investigations conformed to the principles outlined in the Declaration of Helsinki and were performed with permission by the responsible Medical Ethics Committee of Xiangya Hospital, Central South University, China.

## Supplementary Material

Supplementary Table 1

Supplementary Table 2

Supplementary Table 3
