# Supplementary material for: Quantitative analysis of the human ovarian carcinoma mitochondrial phosphoproteome
Source: Aging (Albany NY). 2019 Aug 22;11(16):6449–68. doi: 10.18632/aging.102199 (PMC6738437; doi:10.18632/aging.102199)
Supplement: Supplementary Table 2 [file aging-11-102199-s002.pdf]

Supplementary Table 2. The protein–protein interaction (PPI) network of 67 identified mitochondrial phosphoproteins.

Node 1	Node 2	Coexpression	Combined_score
RPLP0	RPLP2	0.979	0.999
PTGES3	HSP90AA1	0.650	0.999
TOMM20	TOMM22	0.319	0.998
EIF2S2	EIF2B5	0.132	0.997
PRKACA	PRKAR2A	0.355	0.997
TOMM20	VDAC1	0.206	0.994
HSPD1	HSP90AA1	0.917	0.991
VDAC2	VDAC1	0.555	0.991
MYH10	MYH9	0.125	0.984
VDAC3	VDAC1	0.165	0.981
CAV1	HSP90AA1	0.000	0.977
VDAC2	TOMM20	0.188	0.973
VDAC3	TOMM20	0.206	0.971
EIF4G3	EIF2S2	0.089	0.962
HSPD1	VDAC1	0.219	0.960
HSPD1	TOMM20	0.209	0.959
CFL1	HSP90AA1	0.106	0.946
HSPD1	TOMM22	0.124	0.946
RPLP0	EIF2S2	0.409	0.940
EIF2S2	RPLP2	0.324	0.936
HIST1H1C	HIST1H1E	0.348	0.933
HSP90AA1	PRKACA	0.000	0.924
VDAC3	VDAC2	0.115	0.924
RPLP0	PA2G4	0.567	0.910
RPLP0	MRPS16	0.554	0.904
HMGA1	HIST1H1E	0.000	0.901
HMGA1	HIST1H1C	0.000	0.900
CFL1	CFL2	0.048	0.852
HSP90AA1	PSMA3	0.754	0.836
VDAC1	TOMM22	0.125	0.778
RPLP0	PSMA3	0.642	0.778
RPLP0	STT3B	0.064	0.744
RPLP2	PA2G4	0.666	0.743
TOMM20	HSP90AA1	0.108	0.736
VDAC3	TOMM22	0.085	0.735
PA2G4	PSMA3	0.647	0.715
PUS1	MRPS16	0.071	0.696
EIF2S2	PA2G4	0.504	0.669
VDAC2	TOMM22	0.106	0.669
EIF2S2	PSMA3	0.503	0.657
CAV1	EHD2	0.301	0.649
ALG3	STT3B	0.062	0.640
RPLP2	PSMA3	0.633	0.633
ATP2A2	TMX1	0.047	0.628
EIF4G3	EIF2B5	0.129	0.618
PTGES3	HSPD1	0.363	0.597

HSP90AA1	PTPLAD1	0.095	0.556
CAV1	VDAC1	0.000	0.551
CFL1	MYH9	0.091	0.548
CFL1	MYH10	0.064	0.543
TMX1	VDAC1	0.056	0.540
VDAC1	CLN6	0.000	0.529
TMX1	VDAC2	0.056	0.522
HSPD1	PSMA3	0.440	0.522
HSP90AA1	MYH9	0.061	0.521
CFL1	RPLP2	0.167	0.520
HSP90AA1	VDAC1	0.119	0.516
XRN2	U2AF2	0.126	0.514
MYH10	CFL2	0.064	0.512
MYH10	DSTN	0.084	0.498
RPLP0	VDAC3	0.189	0.489
VDAC3	TMX1	0.056	0.489
CTNNA1	PKP2	0.109	0.484
HSP90AA1	PA2G4	0.398	0.480
MYH10	HSP90AA1	0.087	0.479
PTGES3	PA2G4	0.453	0.478
BNIP3L	VDAC1	0.050	0.476
EIF2S2	HDGF	0.084	0.476
OSBP	RMDN3	0.000	0.473
RPLP0	HSP90AA1	0.291	0.465
BZW1	EIF2S2	0.233	0.465
TOMM20	RMDN3	0.062	0.464
MARCKS	HSP90AA1	0.000	0.452
HSPD1	FTMT	0.000	0.451
PTGES3	RPLP2	0.424	0.441
VDAC1	RMDN3	0.000	0.440
ALG3	EIF2B5	0.061	0.433
HSPD1	PRKACA	0.062	0.431
HSPD1	VDAC2	0.158	0.422
VDAC3	HSPD1	0.154	0.413
BNIP3L	TOMM20	0.000	0.410
ATP2A2	MYH10	0.108	0.408
TMX1	DSTN	0.084	0.408
DSTN	MYH9	0.063	0.400
