# Supplementary material for: Quantitative analysis of the human ovarian carcinoma mitochondrial phosphoproteome
Source: Aging (Albany NY). 2019 Aug 22;11(16):6449–68. doi: 10.18632/aging.102199 (PMC6738437; doi:10.18632/aging.102199)
Supplement: Supplementary Table 3 [file aging-11-102199-s003.doc]

**Supplementary Table 3. Phosphoproteins identified in mitochondrial samples prepared from human ovarian cancer and control tissues.** * Phosphorylated residue. #: Oxidated Met residue. $: Carbamidomethyl cystine residue.

| **Protein accession** | **Gene name** | **Protein description** | **PSMs** | **Phosphopeptide** | **phosphoSite Probabilities** | **PhosphoSite in protein sequence** | **Ion Score** | **[M+H]+ [Da]** | **Average A (N)** | **Average B (T)** | **Ratio (T/N)** | **t-test p-value** |
| --- | --- | --- | --- | --- | --- | --- | --- | --- | --- | --- | --- | --- |
| A0A024QZN9 | VDAC2 | Voltage dependent anion channel 2 | 8 | LTFDTTFS*PNTGK | 100 | pS140 | 33.9 | 2117.1 | 0.51 | 1.01 | 1.99 | 7.99E-03 |
| A0A0S2Z472 | PRKAR2A | Protein kinase cAMP-dependent type II regulatory subunit alpha | 1 | VADAKGDS*ES*EEDEDLEVPVPSR | 100.0; 100.0 | pS78; pS80 | 23.2 | 3241.5 | 1.73 | 0.38 | 0.22 | 9.18E-03 |
| A0A140TA73 | SNTB2 | Syntrophin beta 2 | 3 | GLGPPS*PPAPPR | 100 | pS95 | 36.1 | 1526.8 | 1.83 | 0.39 | 0.21 | 7.67E-03 |
| A0A1B0GU90 | CLN6 | Ceroid-lipofuscinosis, neuronal 6, late infantile, variant | 1 | HGS*VSADEAAR | 100 | pS7 | 31.4 | 1483.7 | 0.90 | 0.85 | 0.94 | 8.67E-01 |
| A3R0T7 | HIST1H1E | Liver histone H1e | 5 | S*ETAPAAPAAPAPAEK | 98.5 | pS2 | 55.5 | 1904.9 | 2.29 | 0.28 | 0.12 | 1.23E-04 |
| A8K4L6 | VANGL2 | VANGL planar cell polarity protein 2 | 4 | LQSETS*V | 98.4 | pS517 | 33.4 | 1147.6 | 0.99 | 0.76 | 0.77 | 1.90E-01 |
| A8K646 | OSTF1 | cDNA FLJ75699, highly similar to Homo sapiens osteoclast stimulating factor 1, mRNA | 2 | TLSNAEDYLDDEDS*D | 100 | pS213 | 51.1 | 2085.8 | 2.50 | 1.75 | 0.70 | 8.50E-02 |
| A8K769 | SCAMP2 | Secretory carrier membrane protein 2 | 4 | AAS*SAAQGAFQGN | 99 | pS319 | 73.7 | 1563.7 | 0.38 | 1.10 | 2.92 | 1.49E-03 |
| A8K8G0 | HDGF | Hepatoma-derived growth factor | 3 | AGDLLEDS*PK | 100 | pS133 | 39.4 | 1732.9 | 1.21 | 0.62 | 0.51 | 7.66E-04 |
| B0AZV5 | OSTF1 | cDNA FLJ75699, highly similar to Homo sapiens osteoclast stimulating factor 1, mRNA | 9 | GIPLAT*GDT*S*PEPELLPGAPLPPPK | 33.3; 33.3; 33.3 | pT38; pT41;pS42 | 28.7 | 3152.7 |  |  |  |  |
| B3GQS7 | HSPD1 | Heat shock protein family D (Hsp60) member 1(HSPD1) | 8 | TVIIEQSWGS*PK | 100 | pS70 | 49.6 | 2033.1 | 0.48 | 1.11 | 2.34 | 3.45E-03 |
| B3KX39 | ANK1 | Ankyrin 1 | 1 | LGYIS*VTDVLK | 98 | pS89 | 29.5 | 1896.1 | 0.92 | 0.81 | 0.88 | 7.54E-01 |
| B3KXC3 | FTMT | Ferritin | 8 | HTLGDS*DNES | 100 | pS179 | 40.2 | 1458.6 | 0.99 | 0.75 | 0.76 | 8.76E-03 |
| B4DDC6 | PTGES3 | Prostaglandin E synthase 3 | 6 | DWEDDS*DEDMSNFDR | 100 | pS77 | 64.8 | 2259.8 | 1.53 | 0.53 | 0.35 | 1.70E-05 |
|  |  |  | 7 | DWEDDS*DEDM^#^SNFDR | 100 | pS77 | 64.8 | 2275.8 | 1.47 | 0.59 | 0.40 | 1.32E-03 |
| B4DKT9 | CTNNA1 | Catenin alpha 1 | 7 | TPEELDDS*DFETEDFDVR | 100 | pS518 | 69.3 | 2543.1 | 1.00 | 0.78 | 0.78 | 1.19E-04 |
| B4DMA2 | HSP90 | cDNA FLJ54023, highly similar to Heat shock protein HSP 90-beta | 17 | IEDVGS*DEEDDSGk | 100 | pS217 | 60.7 | 2183.0 | 0.99 | 0.79 | 0.80 | 2.83E-02 |
| B4DU62 | EHD2 | cDNA FLJ54154, highly similar to EH-domain-containing protein 2 | 5 | GPDEAM^#^EDGEEGS*DDEAEWVVTK | 100 | pS80 | 60.2 | 3199.4 | 2.57 |  |  |  |
|  |  |  | 3 | GPDEAMEDGEEGS*DDEAEWVVTK | 100 | pS80 | 57.7 | 3183.4 | 2.45 |  |  |  |
| B4DZE1 | EIF4G3 | cDNA FLJ53944, highly similar to Eukaryotic translation initiation factor 4 gamma 3 | 2 | EAEEES*EDN | 100 | pS1072 | 53.7 | 1435.5 | 1.37 | 0.58 | 0.42 | 1.10E-04 |
| B4DZX7 | TXNDC | Thioredoxin domain containing, isoform CRA_b | 3 | VEEEQEADEEDVS*EEEAESK | 100 | pS163 | 86.4 | 2998.3 | 0.65 | 1.04 | 1.61 | 1.13E-01 |
| B4E0B9 | XRN2 | cDNA FLJ54526, highly similar to 5'-3' exoribonuclease 2 (EC 3.1.11.-) | 1 | KAEDS*DS*EPEPEDNVR | 100.0; 100.0 | pS423; pS425 | 31.3 | 2585.1 | 0.94 | 0.80 | 0.86 | 9.16E-02 |
| B8QGS9 | PKP2 | Plakophilin 2 | 5 | LEIS*PDS*S*PER | 100.0; 50.0; 50.0 | pS151; pS154; pS155 | 29.6 | 1693.7 | 0.55 | 1.02 | 1.86 | 1.61E-03 |
| C9JKI3 | CAV1 | Caveolin 1 | 3 | ADELS*EK | 100 | pS6 | 44.5 | 1217.6 | 2.34 | 0.06 | 0.03 | 8.07E-07 |
| E5RIA0 | SORBS3 | Sorbin and SH3 domain containing 3 | 2 | ADGGS*PFLGR | 100 | pS6 | 42.3 | 1098.5 |  |  |  |  |
| E9PCT5 | CAV1 | Caveolin 1 | 5 | AMADELS*EK | 100 | pS26 | 39.5 | 1681.8 | 2.27 | 0.15 | 0.06 | 9.35E-09 |
|  |  |  | 3 | AM^#^ADELS*EK | 100 | pS26 | 38.4 | 1697.8 | 2.34 | 0.08 | 0.03 | 3.50E-05 |
| E9PS23 | CFL1 | Cofilin 1 | 7 | AS*GVAVSDGVIK | 100 | pS3 | 58.0 | 1528.8 | 1.64 | 0.47 | 0.29 | 8.25E-05 |
| F5GXU9 | BCKDHA | Branched chain keto acid dehydrogenase E1, alpha polypeptide | 3 | S*VDEVNYWDK | 100 | pS318 | 46.3 | 1942.9 | 1.28 | 0.62 | 0.49 | 8.49E-04 |
| F5H168 | PUS1 | Pseudouridylate synthase 1 | 2 | VPSPLEGS*EGDGDT*D | 100.0; 100.0 | pS127;pT133 | 29.5 | 1938.8 | 0.50 | 1.11 | 2.20 | 1.67E-03 |
|  |  |  | 5 | VPSPLEGSEGDGDT*D | 100 | pT133 | 57.5 | 1858.8 | 0.43 | 1.08 | 2.52 | 2.47E-03 |
| F6RFD5 | DSTN | Destrin, actin depolymerizing factor | 6 | AS*GVQVADEVC^$^R | 100 | pS3 | 69.8 | 1412.6 |  |  |  |  |
| F8VR77 | PA2G4 | Proliferation-associated 2G4 | 3 | S*GEDEQQEQTIAEDLVVTK | 100 | pS2 | 54.8 | 2545.2 | 1.92 | 0.75 | 0.39 | 7.45E-04 |
| F8W1K8 | RPLP0 | Ribosomal protein lateral stalk subunit P0 | 2 | VEAKEES*EES*DEDMGFGLFD | 100.0; 100.0 | pS94; pS97 | 45.6 | 3031.3 | 1.85 | 0.32 | 0.17 | 1.96E-05 |
|  |  |  | 6 | VEAKEES*EES*DEDM^#^GFGLFD | 100.0; 100.0 | pS94; pS97 | 51.7 | 3047.3 | 1.81 | 0.35 | 0.20 | 3.07E-04 |
| F8WF93 | ALG3 | ALG3, alpha-1,3- mannosyltransferase | 4 | SGS*AAQAEGLC^$^K | 99.1 | pS13 | 46.3 | 1866.9 | 0.52 | 1.12 | 2.16 | 2.89E-03 |
| G1UI26 | NBAS | Neuroblastoma amplified sequence | 6 | AGEEDEGEEDS*DS*DY*EISAK | 50.8; 50.8; 98.3 | pS473; pS475; pY477 | 44.6 | 2943.2 | 0.72 | 1.11 | 1.53 | 7.95E-02 |
| G3V2U0 | CFL2 | Cofilin 2 | 1 | AS*GVTVNDEVIK | 100 | pS3 | 36.9 | 1657.8 | 2.48 |  |  |  |
| H0YBC7 | BNIP3L | BCL2 interacting protein 3 like | 1 | DHS*S*QS*EEEVVEGEK | 100.0; 100.0; 100.0 | pS95; pS96; pS98 | 30.0 | 2537.0 | 1.60 | 0.70 | 0.44 | 3.49E-01 |
| H0YDD8 | RPLP2 | Ribosomal protein lateral stalk subunit P2(RPLP2) | 16 | KEES*EES*DDDM^#^GFGLFD | 100.0; 100.0 | pS79; pS82 | 44.7 | 2734.1 | 1.77 | 0.37 | 0.21 | 6.46E-06 |
|  |  |  | 8 | KEES*EES*DDDMGFGLFD | 100.0; 100.0 | pS79; pS82 | 59.9 | 2718.1 | 1.91 | 0.30 | 0.16 | 9.02E-06 |
| H0YJ03 | PSMA3 | Proteasome subunit alpha 3 | 7 | ESLKEEDES*DDDNM^#^ | 100 | pS78 | 44.3 | 2360.0 | 1.24 | 0.62 | 0.50 | 4.95E-03 |
| H0YNE5 | RMDN3 | Regulator of microtubule dynamics 3 | 8 | SQS*LPNSLDYTQTSDPGR | 99.9 | pS46 | 45.9 | 2350.1 | 0.95 | 1.08 | 1.14 | 3.98E-01 |
| H7C5W9 | ATP2A2 | ATPase sarcoplasmic/endoplasmic reticulum Ca2+ transporting 2 | 3 | EFDELNPS*AQR | 100 | pS554 | 38.1 | 1689.8 | 1.10 | 0.76 | 0.69 | 2.40E-02 |
| M0QYQ9 | U2AF2 | U2 small nuclear RNA auxiliary factor 2 | 2 | S*DFDEFER | 100 | pS1 | 39.0 | 1166.4 |  |  |  |  |
| O00264 | PGRMC1 | Progesterone receptor membrane component 1 | 2 | EGEEPTVYS*DEEEPK | 97.9 | pS181 | 39.1 | 2426.1 | 0.46 | 1.05 | 2.30 | 4.22E-03 |
|  |  |  | 48 | GDQPAASGDS*DDDEPPPLPR | 100 | pS57 | 105.0 | 2420.1 | 0.86 | 0.88 | 1.02 | 6.25E-01 |
| O75475 | PSIP1 | PC4 and SFRS1 interacting protein 1 | 5 | TGVTS*T*S*DS*EEEGDDQEGEK | 49.0; 49.0; 50.0; 50.0 | pS271; pT272; pS273; pS275 | 60.6 | 2868.2 | 1.55 | 0.45 | 0.29 | 9.76E-03 |
| P16403 | HIST1H1C | Histone cluster 1 H1 family member c | 10 | S*ETAPAAPAAAPPAEK | 98.3 | pS2 | 49.6 | 1904.9 | 2.36 | 0.34 | 0.14 | 4.62E-03 |
| P21796 | VDAC1 | Voltage dependent anion channel 1 | 9 | LTFDSSFS*PNTGK | 100 | pS104 | 56.6 | 2089.0 | 0.42 | 1.09 | 2.63 | 1.58E-04 |
| P22059 | OSBP | Oxysterol binding protein | 4 | MLAES*DES*GDEESVSQTDK | 100.0; 100.0 | pS190; pS193 | 47.5 | 2825.2 | 1.86 | 0.54 | 0.29 | 2.49E-02 |
|  |  |  | 1 | M^#^LAES*DES*GDEESVSQTDK | 100.0; 100.0 | pS190;pS193 | 24.1 | 2841.2 | 1.42 | 0.84 | 0.59 | 1.05E-01 |
| P35579 | MYH9 | Myosin heavy chain 9 | 14 | GAGDGS*DEEVDGK | 100 | pS1943 | 43.5 | 1923.9 | 0.70 | 0.95 | 1.36 | 1.86E-03 |
|  |  |  | 7 | KGAGDGS*DEEVDGK | 100 | pS1943 | 40.2 | 2356.2 | 0.67 | 0.93 | 1.40 | 7.71E-03 |
| P35580 | MYH10 | Myosin heavy chain 10 | 3 | QLHLEGAS*LELS*DDDTESK | 100.0; 100.0 | pS1952; pS1956 | 65.2 | 2855.3 | 0.57 | 1.00 | 1.77 | 3.99E-02 |
|  |  |  | 4 | QLHLEGASLELS*DDDTESK | 100 | pS1956 | 55.6 | 2775.3 | 0.99 | 0.80 | 0.80 | 3.68E-01 |
|  |  |  | 4 | TSDVNETQPPQS*E | 100 | pS1975 | 30.9 | 1815.8 | 1.05 | 0.86 | 0.82 | 3.80E-01 |
| Q13144 | EIF2B5 | Eukaryotic translation initiation factor 2B subunit epsilon | 2 | EAEEES*S*EDD | 100.0; 100.0 | pS717; pS718 | 65.7 | 1603.5 | 1.75 | 0.54 | 0.31 | 1.79E-03 |
| Q13442 | PDAP1 | PDGFA associated protein 1 | 1 | SLDS*DES*EDEEDDYQQK | 99.9; 100.0 | pS60; pS63 | 28.5 | 2800.1 | 1.32 | 0.60 | 0.45 | 1.85E-03 |
| Q15136 | KIN27 | Protein kinase A-alpha (Fragment) | 9 | T*WT*LC^$^GTPEYLAPEIILSK | 50.0; 50.0 | pT179; pT181 | 32.5 | 2880.5 | 1.66 | 0.64 | 0.38 | 1.10E-04 |
| Q15388 | TOMM20 | Translocase of outer mitochondrial membrane 20 | 5 | IVS*AQSLAEDDVE | 100 | pS135 | 62.2 | 1759.8 | 0.45 | 1.16 | 2.56 | 8.46E-05 |
|  |  |  | 4 | IVS*AQS*LAEDDVE | 100.0; 100.0 | pS135; pS138 | 68.8 | 1839.8 | 0.35 | 1.24 | 3.54 | 1.36E-04 |
| Q1XBU7 |  | Aging-associated protein 14a | 3 | ADEPS*S*EES*DLEIDK | 100.0; 100.0; 100.0 | pS75; pS76; pS79 | 29.9 | 2512.0 | 1.83 | 0.58 | 0.32 | 1.24E-02 |
| Q3B7I9 | EIF2S2 | Eukaryotic translation initiation factor 2 subunit beta | 3 | S*GDEMIFDPTMSK | 100 | pS2 | 32.7 | 1883.8 | 1.90 | 0.76 | 0.40 | 8.71E-03 |
|  |  |  | 3 | S*GDEM^#^IFDPTMSK | 100 | pS2 | 46.2 | 1899.8 | 4.73 | 1.98 | 0.42 | 4.97E-02 |
|  |  |  | 3 | S*GDEM^#^IFDPTM^#^SK | 100 | pS2 | 47.7 | 1915.8 | 1.48 | 0.84 | 0.57 | 9.03E-02 |
| Q3LIC9 | BZW1 | Basic leucine zipper and W2 domains 1 | 1 | NAEEES*ES*EAEEGD | 100.0; 100.0 | pS29; pS31 | 61.4 | 1988.7 |  |  |  |  |
| Q4VBX8 | ANKIB1 | Ankyrin repeat and IBR domain containing 1 | 1 | GVAPADS*PEAPR | 100 | pS89 | 30.0 | 1550.7 | 0.87 | 0.84 | 0.96 | 9.24E-01 |
| Q53GB0 | TOM22 | Mitochondrial import receptor Tom22 variant (Fragment) | 13 | AAAVAAAGAGEPQS*PDELLPK | 100 | pS15 | 64.6 | 2389.2 | 0.35 | 1.16 | 3.31 | 1.29E-05 |
| Q5CZB5 | DKFZp686M0430 | Putative uncharacterized protein DKFZp686M0430 | 3 | RGS*S*S*DEEGGPK | 100.0; 100.0;100.0 | pS591; pS592; pS593 | 29.8 | 2053.8 | 2.28 | 0.07 | 0.03 | 9.80E-06 |
| Q5T6U8 | HMGA1 | High mobility group AT-hook 1 | 3 | EEEEGISQES*S*EEEQ | 99.9; 100.0 | pS92; pS93 | 96.4 | 2202.8 | 0.59 | 1.20 | 2.02 | 1.71E-03 |
|  |  |  | 1 | EEEEGIS*QES*S*EEEQ | 100.0; 100.0; 100.0 | pS88; pS92; pS93 | 41.1 | 2282.8 |  |  |  |  |
|  |  |  | 3 | EEEEGIS*QES*S*EEEQ | 33.3; 33.3; 33.3 | pS88; pS92; pS93 | 25.8 | 2122.8 |  | 2.03 |  |  |
| Q6NVI1 | MARCKS | Myristoylated alanine rich protein kinase C substrate | 4 | GEPAAAAAPEAGAS*PVEK | 100 | pS101 | 30.9 | 2311.2 | 1.99 | 0.96 | 0.48 | 5.33E-02 |
| Q8TCJ2 | STT3B | STT3B, catalytic subunit of the oligosaccharyltransferase complex | 17 | ENPPVEDS*S*DEDDKR | 100.0; 100.0 | pS498; pS499 | 46.9 | 2500.1 | 1.01 | 0.77 | 0.77 | 1.14E-02 |
|  |  |  | 5 | ENPPVEDS*S*DEDDKR | 50.0; 50.0 | pS498; pS499 | 25.9 | 2420.1 | 1.13 | 0.71 | 0.63 | 5.30E-02 |
| Q8WVF3 | RAB28 | RAB28, member RAS oncogene family | 2 | S*DS*EEESQDR | 100.0; 100.0 | pS2; pS4 | 39.4 | 1383.4 |  |  |  |  |
| Q96QR8 | PURB | Purine rich element binding protein B | 2 | ADGDS*GSER | 100 | pS6 | 29.4 | 1015.3 |  |  |  |  |
| Q9BSY0 | PTDSS1 | Phosphatidylserine synthase 1 | 3 | HAGNNESHS*S*R | 100.0; 100.0 | pS312; pS313 | 61.5 | 1659.7 | 2.24 | 0.20 | 0.09 | 4.70E-03 |
| Q9NWC5 | TMEM45A | Transmembrane protein 45A | 1 | EQES*EEEM^#^ | 100 | pS271 | 36.8 | 1410.5 | 0.76 | 0.89 | 1.17 | 2.48E-01 |
| Q9P035 | HACD3 | 3-hydroxyacyl-CoA dehydratase 3 | 7 | WLDES*DAEMELR | 100 | pS114 | 41.1 | 1877.8 | 1.07 | 0.76 | 0.71 | 7.04E-03 |
|  |  |  | 15 | WLDES*DAEM^#^ELR | 100 | pS114 | 21.7 | 1893.8 | 0.77 | 0.90 | 1.18 | 2.76E-02 |
| Q9UHS8 | SQOR | Sulfide quinone oxidoreductase | 3 | YPNVFGIGDC^$^TNLPTS*K | 99.9 | pS286 | 38.1 | 2571.3 | 0.17 | 1.23 | 7.20 | 1.46E-03 |
| Q9Y277 | VDAC3 | Voltage dependent anion channel 3 | 1 | C^$^NT*PTYC^$^DLGK | 100 | pT6 | 47.8 | 1754.7 |  | 1.28 |  |  |
| Q9Y3D3 | MRPS16 | Mitochondrial ribosomal protein S16 | 3 | TDAEAT*DTEATET | 100 | pT130 | 41.0 | 1738.7 |  | 1.53 |  |  |
